# Massive Ascites Following Robot-Assisted Radical Prostatectomy and Extended Pelvic Lymph Node Dissection: A Case Report

**DOI:** 10.1089/cren.2018.0080

**Published:** 2019-12-02

**Authors:** Shyaw Ahmed, Greg Shaw, Omar AlKadhi

**Affiliations:** Department of Urology, University College London Hospitals NHS Foundation Trust, London, United Kingdom.

**Keywords:** massive ascites, robot-assisted radical prostatectomy, lipidiol lymphangiogram

## Abstract

***Background:*** Lymph leakage is regarded as one of the rare complications of major abdominal, pelvic, and thoracic surgeries. Lymphangiogram seems to be the principal diagnostic modality. Management strategies that have been shown in the literature range from conservative measures to surgical exploration. However, the rarity and diversity in the presentation of this complication have attributed to the lack of consensus and guideline on its management.

***Case Presentation:*** A 49-year-old obese man with prostate-specific antigen of 10 and preoperative Gleason score of 8 prostate cancer and initial staging of T_3_N_0_M_0_ has undergone robot-assisted radical prostatectomy and extended pelvic lymph node dissection with unilateral nerve sparing. Our patient was admitted with significant ascites on day 14 postoperative, which was confirmed on CT abdomen and initially managed with nutritional support and percutaneous drainage. A lipidiol lymphangiogram demonstrated lymphatic leakage near the right external iliac vein. While he was awaiting elective surgical exploration, he has had two further successive admissions with massive ascites, anemia and raised C-reactive protein with acute kidney injury. A laparoscopic exploration was performed with interventional radiology assistance to direct dissection to the site of leak. An abscess cavity was found and excised. The lymphatic leak tailed off to insignificance rapidly thereafter.

***Conclusion:*** Each case of lymphatic leakage seems to require an individualized approach according to the nature and severity of the lymphatic leak and patient condition. Although it is possible that the collection was infected lymphatic fluid, the position of the abscess cavity in proximity to the site where the lipidiol was seen to leak from the lymphatics suggests that it is possible that the lipidiol was the nidus for infection. Either way what is interesting is that the presence of the abscess caused prolonged and profuse lymphatic leakage.

## Introduction

Lymphatic leakage is regarded as one of the rare complications of major abdominal, pelvic, and thoracic surgeries, particularly procedures that involve lymph node dissection.^1^ Lymphangiogram seems to be the principal modality in the diagnosis and identification of the leakage site with occasional therapeutic benefits.^2^ Different modalities of treatment have been suggested in the literature mainly focusing on conservative measures including dietary support and pharmacological treatment such as octeriotides.^3^

## Case Presentation

A 49-year-old obese (body mass index of 35.7 kg/m^2^) man with a background of hypertension, asthma, and well-controlled schizoaffective disorder was referred for robot-assisted radical prostatectomy (RARP). His digital rectal examination was T3 feeling prostate with a prostate-specific antigen of 10 ng/cc and Gleason grade of 8 in three out of six cores (all on right). His staging on MRI was T_3a_N_0_ with likely capsular invasion and bone scan was negative (M0).

He underwent RARP and extended pelvic lymph node dissection with unilateral nerve sparing. A thickened lymphatic tissue (likely a large lymph node) was noted on the right hand side of the bladder and was resected. During the difficult procedure, intraoperative blood loss was 1 L with no major complications. Postoperative finding was uneventful with his drain only draining 200 mL and the patient was discharged on day 2 postoperation.

**FIG. 1.**
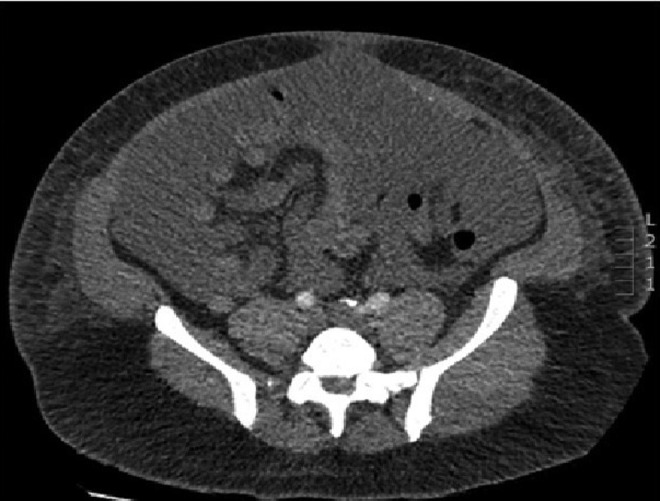
Abdominal CT scan shows massive ascites.

Two weeks later he was admitted with significant ascites, genital and lower limb edema with clear fluid from the surgical wound. CT scan showed gross ascites (Fig. 1). Initially ascites was managed with nutritional support and percutaneous drainage producing ∼2 L of transudate a day for >3 weeks.

Histology analysis from the prostatectomy showed upgrading of Gleason score to 4 + 5 (cribriform and solid nests with central comedo necrosis) with established extensive right posterior extraprostatic extension and involvement of right posterior extraprostatic margin for a maximum circumference of 2 mm. In addition, one of seven lymph nodes from the right pelvis showed metastatic prostate adenocarcinoma (1/7). However, left pelvic lymph nodes revealed no metastatic malignancy (0/5). Therefore, hormonal therapy was started while inpatient and adjuvant radiotherapy was planned.

**FIG. 2.**
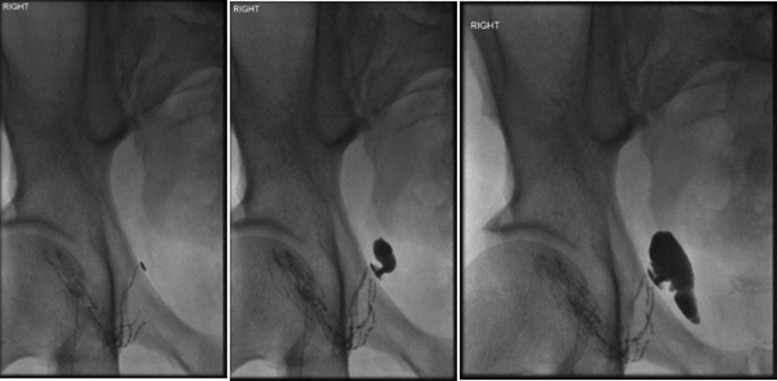
Lymphangiogram; Ultrasound-guided right superficial lymph node puncture. Lipiodol was infused. Leak identified at level of distal external iliac vessels just superior to the superior pubic ramus.

A lipidiol lymphangiogram showed lymphatic leakage near the right external iliac vein (Fig. 2). An initial decrease in drainage prompted discharge. Two weeks later he was readmitted with ascites, which was again drained and elective exploration was planned. However, he was readmitted as an emergency 10 days later with massive ascites, respiratory distress, signs of infection, anemia, and raised C-reactive protein (CRP) with acute kidney injury. He was managed with antibiotics, packed red cell transfusion, and ascites drainage.

Laparoscopic exploration was performed with interventional radiology assistance to direct dissection to the site of the leak. An abscess cavity was found and excised. The lymphatic leak tailed off to insignificance rapidly thereafter.

## Discussion

Lymphatic leakage is a rare but significant sequela of major abdominal and thoracic surgeries that can be debilitating for both patients and health services because of its chronic nature. Our patient has presented with massive transudative ascites that has evidently failed to respond to conservative measures and continue to have lymph leakage and suffer majority of its complications such as malnutrition, anemia, and above all infection.

In our patient, lipidiol lymphangiogram was performed with injection direct into the groin nodes under ultrasound guidance that demonstrated obvious lymphatic leak. Matsumoto et al.^2^ have suggested that early lymphangiogram seems to be a viable option for patients with continuous lymphatic leakage with potential therapeutic value in 89% of cases. However, in this case, it is possible that lipidiol may have acted as a nidus for infection and also it remained in the body for many weeks and mimicking contrast on CT and fluoroscopy.

**FIG. 3.**
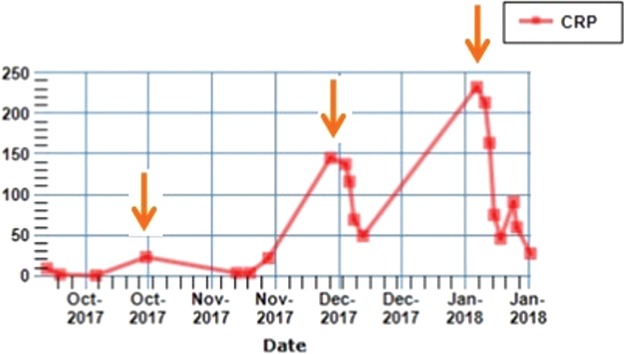
A graph showing CRP curve over time. Note *orange arrows* that correspond to CRP increment with each individual admission. CRP, C-reactive protein.

It is worth noting that with each individual admission, the patient had a rise in CRP, which is shown in Figure 3. This may imply an element of inflammatory reaction and possible superadded infection.

In a case series, four cases of chylous ascites after RARP with extended pelvic lymph node dissection have been reported.^4^ All four cases of prostatectomies responded to conservative measures including draining. Nonetheless, in our situation with massive ascites, the surgical exploration appeared to resolve the problem. Therefore, as concluded by Leibovitch et al.,^3^ each case seems to require an individualized approach according to the nature and severity of the lymphatic leak and patient condition. Moreover, although it is possible that the collection was infected lymphatic fluid, the appearance of the abscess cavity situated at the site where the lipidiol was seen to leak from the lymphatics suggests that it is possible that the lipidiol was the nidus for infection. Either way what is interesting is that the presence of the abscess caused prolonged and profuse lymphatic leakage.

## Abbreviations Used

CRP: C-reactive protein
CT: computed tomography
MRI: magnetic resonance imaging
RARP: robot-assisted radical prostatectomy
